# 2024 Acknowledgment of *mSphere Ad Hoc* Reviewers

**DOI:** 10.1128/msphere.01041-24

**Published:** 2025-01-28

**Authors:** Michael J. Imperiale

**Affiliations:** 1 Department of Microbiology and Immunology, University of Michigan Medical School, Ann Arbor, Michigan, USA

## EDITORIAL

As I started to write this, I thought that I would start off by doing something scholarly. I went to the Merriam-Webster dictionary and looked up “reviewer.” Its definition: one who reviews. Well, yeah, I cannot argue with that. I next consulted the Oxford English Dictionary. It has three definitions, the last of which comes closest to what I was hoping to find: one who criticizes new publications; a writer of reviews. However, I was still unsatisfied because neither of these fully reflects the way we view reviewers at *mSphere*. To us, reviewers are an integral part of the scientific publishing ecosystem, contributing to our goal of helping authors publish the best work that they can. They are not there only to criticize but to be helpful partners to the authors and editors. As an editor, I always read reviews with one major consideration in mind: how can this review help the authors improve what they have written so that our broad readership will appreciate the effort that went into the experiments, conclusions, and interpretations presented in the manuscript?

We all know that reviewing can be a seemingly thankless job, something I discussed in last year’s acknowledgments. But I think that each of us who reviews manuscripts knows that it is our obligation to the research enterprise to contribute, in whatever way we feel comfortable, to the betterment of our collective science. And each of us who is part of this enterprise must be grateful for the work that reviewers do. On behalf of all the senior editors and editors at *mSphere*, I give my sincere thanks to the following individuals, who took the time from November 2023 through November 2024 to help achieve these goals.

Grace Onyukwo Abakpa

Ruth Chingcuangco Abanador

Ghulam Abbas

Shairah Abdul Razak

Jehane Abed

Alaa Abouelfetouh

Sabrina Absalon

Michael Abt

Walter Adams

Zach Adelman

Edwin Ades

Clement Gascua Adu-Gyamfi

Muhammad Afzal

Surya Aggarwal

Mir Alvee Ahmed

Bulbul Ahmed

Tuomas Aivelo

Sarah Alaei

Md Tauqeer Alam

Alexandre Alanio

Ashraf Al Ashhab

Rosie Alegado

Pau Alfonso i Comos

Inas Mohamed Alhudiri

Ifey Alio

Thibault Allain

Kyle Allison

Jontana Allkja

Suleiman Al Obeid

Beatriz Alvarez

Samat Amat

Heather Amato

Sushma-Vadiraj Ambekar

Rocio Amorín De Hegedüs

Rosemary Aogo

Gabriele Arcari

Linda Archambault

Edwin Armitage

Paul Arnaboldi

Karen Arndt

Benjamin Aroeti

Appudurai Arulkanthan

Vaithilingaraja Arumugaswami

Walter Atwood

Jennifer Auchtung

Jacques Augenstreich

Leonardo Augusto

Sean Avedissian

Deepika Awasthi

Minimol Ayyappan

Faustus Azerigyik

Taeok Bae

Yong-Sun Bahn

Sakcham Bairoliya

Lauren Bakaletz

Steven Baker

Cynthia Baldwin

Jimmy Ballard

Jeffrey Banas

Arinjay Banerjee

Ehud Banin

Matthew Barber

Bridget Barker

Samuel Barnett

Ravi Pratap Barnwal

Antonio Barragan

Patricia Andrea Barrera

Michael Barrett

Jason Bartz

Martine Bassilana

Christine Marie Bassis

Mariano Battistuzzi

Inga Bazukyan

Clifford Beall

Michael Becker

Kathleen Beilsmith

Graham John Belsham

Emily Bendall

Rudolf Beran

Albert Berghuis

Celia Betencourt

Omolola Comfort Betiku

Purnima Bhanot

Disha Bhattacharjee

Pablo Bifani

Blake Billmyre

Lyn-Marie Birkholtz

Karishma Bisht

Basanta Kumar Biswal

Johanna Björkroth

Lexie Christine Blalock

Matthew Blango

David C. Bloom

Patricia Pringle Bloom

Karl Boehme

Joseph Boll

Paul Bollyky

Jennifer Bomberger

Germán Bonilla-Rosso

Thomas Boren

Andrew Borman

Ana Caroline Nunes Botelho

Elsa Bou Ghanem

Steeve Boulant

Prosper Boyaka

John Dallas Boyce

Jon Boyle

Doug Brackney

Spencer Brady

Alexandra C. Brand

Hans Breeuwer

Shaun R. Brinsmade

Sylvain Brisse

Robert Britton

Cara Brook

Christopher Brooke

Hilary Browne

Iris Bruchhaus

Harald Bruessow

John H. Brumell

Sascha Brunke

Vincent Michael Bruno

Jessica Michelle Bryant

Mary Bryk

Clifton Bueno De Mesquita

Laura Burrack

Geraldine Butler

Mariana Byndloss

Nicole Bzdyl

Ben Callahan

Cristina Calvo

Jodi Camberg

Kenneth Campellone

Xin Cao

Nicholas Carbonetti

Hannah Carey

Jason Carlyon

Andrés Carrazco Montalvo

Michael Carruthers

Santiago Castillo-Ramírez

Nuno Cerca

Benjamin Chadwick

Subbaiah Chalivendra

Mathias Chamaillard

Kitti Wing Ki Chan

Kuan Rong Chan

Jiasong Chang

Siriruk Changrob

Daniel Charlier

Subhadeep Chatterjee

Minu Chaudhuri

Neeraj Kumar Chauhan

Francisco Chavez

Chia Wei Cheah

Changbin Chen

Grace Chen

Jioinglin Chen

Miguel Angel Chiurillo

Michaelle Chojnacki

Patchanee Chootong

Chia-Ching Chu

Nicholas Cianciotto

Sarah Clark

Anna Ruth Cliffe

Stephen Cole

Sarah Comstock

Sean Conlan

Alfred Cortés

Richard Costa Polveiro

Timothy Cover

Robert Cramer

Hannah Creager

Cristian Crisan

Shaun Trent Cross

Sean Crosson

Bo Cui

Paul Cullen

Kyle Cunningham

Chiara Curra

Xiaoxia Dai

Heath Damron

Ajai Dandekar

Joshua Daniels

Patrick D'Aoust

Stuart Dashper

Raymond James Dattwyler

Diwakar Davar

Robert Davey

Bryan Davies

Keith Davies

Ryan Dustin Davis

Suzanne Dawid

Neta Dean

Anusuya Debnath

Jean-Winoc Decousser

George Deepe

Elizabeth De Gaspari

Christopher Luis De Graffenried

Piet De Groot

Roberto De Guzman

Eric Delwart

Erick Denamur

Xuming Deng

Nicole De Nisco

Daniel De Oliveira Patricio

Jaap Deroode

Jigar Desai

Corrella Detweiler

Diane Dieuliis

Eunsoo Do

Karen Dobos

Roberto Docampo

Tamara Doering

Chun-Liu Dong

Tao Dong

Yan Dong Niu

Katherine Shea Dowdell

Karl Drlica

Chen Du

Khrystyne N. Duddleston

Edward Dudley

Micah Dunthorn

Zoe Dyson

Leo Eberl

Ester Eckert

Tom Edlind

Andrew Edwards

Hanareia Ehau-Taumaunu

Amir Elalouf

Melissa Ellermann

Jeremy Ellermeier

Mindy Anne Engevik

Markus Engstler

Alexander Ensminger

Jesse Erasmus

Mehmet Erinmez

Shankar Esaki Muthu

Emilie Esnault

Manuel Espinosa-Urgel

Ronald Drew Etheridge

Tom Evans

Sarah Elisabeth Ewald

Kate Excoffon

Natalie Giannelli Exum

Keke Fairfax

Yanhua Fan

Ferric Fang

Ying Fang

Herman Favoreel

Zhixin Feng

Christopher Fenno

Jean-Baptiste Ferdy

Kenneth Fields

Daniel Figeys

Scott Filler

David Fitzpatrick

Joshua Fletcher

Mike Flint

Enrique Flores

Jarrod Fortwendel

Derek Foster

Elizabeth Fozo

Jeffrey Freiberg

Claudia Freire

Karine Frenal

Naoki Fukuma

Mathis Funk

Joao Gabriel Silva Souza

Matthias Gaestel

Yunpeng Gai

Sukirth Ganesan

Michael Ganzle

Chao Gao

Lujuan Gao

Melina Garcia Guizzo

Uma Shankar Gautam

Christian Gauthier

Michael Gebhardt

Martin Gengenbacher

Sarah George

Susanne Gerber

Aleeza Gerstein

Shimaa Ghazal

David Gillum

Matthew Gilmour

Philippe Glaser

Katharina Goerlich

Alyssa Golden

Richard Gomer

Angela Gomez-Simmonds

Vijay Gondil

Jun Gong

Jose Reyes Gonzalez-Galaviz

Andrew Gorringe

Ria Goswami

Jerome Govin

Katherine Graham

David Grainger

Lone Gram

Chris Grant

Urs Greber

Keith Green

Casey Gries

Kirsten Grond

Gigi Kwik Gronvall

Marc-Jan Gubbels

Bronwyn Gunn

Peter Gyarmati

Hubertus Haas

Daisuke Hagiwara

Sofia Hain

Mark Hall

Rebecca Hall

Larry Halverson

Siegfried Hapfelmeier

Elizabeth Hartland

Erin Harvey

Karl Hassan

Florencia Haurat

Wenqi He

Zhili He

Aoife Heaslip

Tyler Helmann

Zheng Heping

Sander Herfst

Lara Herrero

Zoe Hilbert

Kent Hill

Markus Hilty

Deborah Hinton

Yuu Hirose

Jeffrey Holloman

Geoffrey Holm

Pascal Hols

Jennifer Honda

Isobella Honeyborne

Peiying Hong

Xiao-Yue Hong

Fan Hongjie

Alexander Horswill

Edith Houben

Galadriel Astra Hovel-Miner

Kate Joanne Howell

Lesley Hoyles

Haijiang Huang

Manning Huang

Shi Huang

Weiwei Huang

Casey Hubert

Gary Huffnagle

Katherine Hummels

Jillian Hurst

Marion Hutinel

Tuanh Huynh

Kevin Hybiske

Rodrigo Ibarra Chavez

Carolyn Ibberson

Alexander Ignatov

Asparouh Iliev

Tomohiro Inaba

Ralph Isberg

Jerilyn Izac

Sylwia Jafra

Sumita Jain

Victoria Jeffers

Ann Jerse

Travis Jewett

Hongpeng Jia

Hongchen Jiang

Yong Jiang

Ying Jing

Jörgen Johansson

Jojy John

Regina Joice Cordy

Chet Joyner

Timothy Julian

Won Hee Jung

Pilar Junier

Sreenath K

David Kadosh

Barbara Kahl

Markus Kainulainen

Jessica Kajfasz

Pallavi Kakade

Lindsay Kalan

Alexis Kalergis

Nicolai Kallscheuer

Manabu Kanno

Justin Kaspar

Hiromi Kato

Hangjun Ke

Thomas Kehl-Fie

William Kelley

Alyson Kelvin

Linda Kenney

Fazlurrahman Khan

Nicolas Kieffer

Anders Kiledal

Byoung Sik Kim

Peter Kima

Jason Kindrachuk

Gary King

Chih-Yen King

Georgios Kitsios

Nicholas Kiulia

Michiel Kleerebezem

Michael Klemba

Laura Knoll

Chunkyu Ko

Kathrin Kober-Rychli

Larry Kociolek

Julia Ruth Köhler

Noah Kojima

Hisayuki Komaki

Hitoshi Komatsuzawa

James Konopka

Asja Korajkic

Daniel Kornitzer

Konstantin Korotkov

Natalia Korotkova

Loly Kotta-Loizou

Akihiro Koyama

Lukasz Kozubowski

Abby Kroken

James Kronstad

Damian Krysan

Sarah Kuehne

Carol Kumamoto

Anand Kumar

Awanish Kumar

Dhiraj Kumar

Sanjeev Kumar

Santosh Kumar

Samarchith Kurup

Hiroyuki Kusada

Hyun Jin Kwun

Maisem Laabei

Maria-Halima Laaberki

Benjamin Lamp

Noelia Lander

Stephanie Langel

Pawel Laniewski

Timothy Lapara

Johan Larsbrink

Beth Lazazzera

Emily Ledgerwood

James Leduc

Chia Lee

Christine Lee

Elliot Michael Lee

Kangseok Lee

Kyung-Tae Lee

Sang Jun Lee

Vincent Lee

Daniella Lefteri

Mckenzie Lehman

David Leitsch

Danielle Lemay

Jose Lemme Dumit

Joy Leng

Pok Man Leung

Gina Lewin

Feng Li

Fengqiao Li

Hongjun Li

Huan Li

Mingkun Li

Renfeng Li

Shuzhen Li

Yuchun Li

Guodong Liang

Renxing Liang

Julian Liber

Tami Lieberman

Shen Jean Lim

Bruno Lima

Romana Limberger

Dominique Limoli

Jianfeng Lin

Lasse Lindahl

Haoping Liu

Huiquan Liu

Jun Liu

Monica Liu

Qingyun Liu

Yong-Xin Liu

Malina Loeher

Lotus Lofgren

Latania Logan

Catherine Logue

Torey Looft

Jesus Lopez

Américo Harry López-Yglesias

Jeffrey Lorch

Alex Loukas

Andrew Lee Lovering

Anice Lowen

Xiaojie Lu

Kuan-Yi Lu

Ling Lu

Micah Luftig

Bo Luo

Joseph Lutkenhaus

Meghan Lyman

Joshua Mark Lyte

Jingyun Ma

Matthias Machner

Milton Maciel

Hiten Madhani

Melissa Maginnis

Michael J. Mahan

Ashish Malik

Elizabeth Mallott

Anita Manogaran

Michael Mansour

William Margolin

Julia Martin

Adam Matson

Jyl Matson

Jere McBride

Beth McCormick

John McCormick

John McCrone

Clif McKee

Debbie McKenzie

Anna McLoon

Joel McManus

Joseph B. McPhee

Bob Meek

Ran Mei

Jay Mellies

Vineet Menachery

Jianghong Meng

Douglas Scott Merrell

John Scott Meschke

Ilhem Messaoudi

Ella Meumann

Sarah Mielke

Antonella Migliaccio

Laura Mike

Piers Millett

Samuel Miravet-Verde

Bibhuti Mishra

Ruchir Mishra

Dominique Missiakas

Angela Marie Mitchell

Michelle Momany

Kyung Moon

Julie Moore

Robert Moore

Hector Mora-Montes

Gary Moran

Cristiano Gallina Moreira

Andrea Moreno Switt

Hiroshi Mori

Joachim Morschhäuser

Paul Moss

Erick Motta

W. Scott Moye-Rowley

Juan Mucci

Samantha Muccilli

Sagori Mukhopadhyay

Carol Munro

Erik Munson

Courtney Murdock

Saisubramanian Nagarajan

Moon Nahm

Yu Nakajima

Takashi Narihiro

Paola Navarrete

Daniel Neill

Ashley Nelson

Jeniel Nett

Hayley Newton

Renee Nicole Ng

Marisa Fabiana Nicolás

Matthew Nilles

Tomoki Nishioka

Cecilia Noecker

Douglas Norris

Cinthia Nuñez

Dennis Nurjadi

Charles Nyagupe

Joshua Obar

David O'Callaghan

Missiani Ochwoto

Tamara O'Connor

David O'Dwyer

Toru Okamoto

Philipp Olias

Macy Olson-Wood

Eugenia Ziying Ong

Andrés Opazo-Capurro

Aharon Oren

Carlos Orihuela

Christina Orrú

Morwan Osman

Kyla Ost

Ana Beatriz Furlanetto Pacheco

Jhang Ho Pak

Glen Palmer

Tracy Palmer

Wei Pan

Sasmita Panda

John Panepinto

Gitika Panicker

Pinaki Panigrahi

Sneh Lata Panwar

Daniel Paredes-Sabja

Laila Pamela Partida-Martinez

Sally Partridge

Edward Patterson

Atmika Paudel

Miguel Penalva

Christian Pérez

Brian Peters

Jason Peters

William Petri

Aleksandra Petrovic Fabijan

Yvonne Pfeifer

Janessa Pickering

Kurt Henry Piepenbrink

Margaret Pitt

Roman Pogranichniy

Laurent Poirel

Francesco Polazzo

Grace Pold

Neha Potnis

Elijah Powell

Suzette Priola

Charles Puerner

Tanel Punga

Catherine Putonti

Xin Qian

Yongkang Qiao

Janet Quinn

Lilliana Radoshevich

Kathryn Rahlwes

Vemulapalli Ramesh

Xiancai Rao

Chad Rappleye

Jeremy Ratcliff

Jason Rauceo

Ratna Ray

Kasie Raymann

Laurie Read

Michael Reese

Aaron Reinke

Ryan Relich

David Relman

Federico Rey

Lisa Reynolds

Christopher Aaron Rice

Nicole Ricker

Laure Ries

Sebastián Riquelme

Erle Robertson

Joana Rocha-Pereira

Nicole Rockey

Marcio Rodrigues

Juan Carlos Rodriguez

Christian Röhrig

Monica Rolando

Ute Römling

Jason Rosch

Nuria Ros-Rocher

Sarah Elizabeth Rowe

Hang Ruan

Brian Russo

Jeffrey Ryback

Marat Sadykov

Suzana Pinto Salcedo

Steven Salzberg

John Samuelson

Jonas Sandbrink

Ranjodh Sandhu

Dominique Sanglard

Sarah Sansom

Felipe Santiago-Tirado

Joana Santos

Manuela Santos

Thomas Sauters

Gary Sawers

Karen Scanlon

Joy Scaria

Maria Scaturro

Patrick Schlievert

Patrick D. Schloss

Jonathan E. Schmitz

Thamarai Schneiders

Hildgund Schrempf

Audrey Schuetz

Antony Schwartz

Oliver Schwengers

Kelly Seaton

Patrick Secor

Anna Maria Seekatz

Julie Segre

Rene Segura

Adnane Sellam

Chao Shan

Robert Shanks

Jesse Shapiro

Ashu Sharma

Itai Sharon

Allyson Shea

Michael Sheedlo

Zhangqi Shen

Ran Shi

Xingdong Shi

Shwetha Shivaprasad

Sloan Siegrist

Ritam Sinha

Ciaran Skerry

Svetoslav Nanev Slavov

Thomas John Smith

Will Smith

Wiep Klaas Smits

Jinxing Song

Qinye Song

Joseph Sorg

Moriah Sparza

Grace Spatafora

Petra Spidlova

Chelsey Spriggs

Chittur Srikanth

Meghan Starolis

Gabriel Starrett

Christine Stauber

Laura St. Clair

Joerg Steinmann

Paul Stodghill

Marc Strous

Jörg Stülke

Jian-Qiang Su

Sandra Suescun

Shinya Sugimoto

Michael Suits

Prawat Sukphun

Guorong Sun

Xingmin Sun

Yankuo Sun

Yanni Sun

Dongchang Sun

Komwit Surachat

Michael Surette

Colin Sutherland

Akihiko Suzuki

Shunya Suzuki

Staffan Svärd

Marc Swidergall

Edward Swords

Toru Takeshita

Xu Tan

Haixu Tang

Akio Tani

Marcus Melo Teixeira

Hasan Tekedar

Chaitanya Tellapragada

Lesly Temesvari

Doron Teper

Kevin Tetteh

Kevin Theis

Carissa Michelle Thomas

Vinai Chittezham Thomas

Justin Thornton

Lance Thurlow

Marc Torrent

German Traglia

Stephen Trent

Ralph Tripp

Tom Troth

Argyro Tsipa

Clement Tsui

Cagla Tukel

Kendra Turk-Kubo

Claire Elizabeth Turner

Gregory Tyrrell

Taher Uddin

Syun-Ichi Urayama

Jane Usher

David Ussery

Miguel Valvano

Laura Vanagas

Russell Vance

Karen Jane Vanderwolf

Patrick Van Dijck

Jan-Peter Van Pijkeren

Trevor Vanschooneveld

Christopher Vassallo

Roberto Vazquez-Munoz

Binu Velayudhan

Daniel Vélez-Ramírez

Katrina Velle

Antony Vincent

Tomeu Viver

Joseph Vogel

Jay Vornhagen

Denis Voronin

Hiep Vu

Bao Vuong

Theresa Maria Wagner

Satoshi Waguri

Nina Wale

Seth Walk

Daniel Wall

Aibing Wang

Chengming Wang

Hui Wang

Jie Wang

Kai Wang

Liang-Chun Wang

Luoyang Wang

Nian Wang

Pandeng Wang

Peng George Wang

Shiwei Wang

Siran Wang

Tian Wang

Xiaolei Wang

Xifeng Wang

Yang Wang

Yifan Wang

Yihui Wang

Keyi Wang

Matthew Wargo

Lauren Waters

Michael Edmund Watson

Scott Weaver

Mary Weber

Min Wei

Yahan Wei

Michael Weigand

Louis Weiss

Stephen Robert Welch

Changjiang Weng

Zhang Wenping

Dawn Wetzel

Robert Wheeler

Amelia White

John Kerr White

Malcolm Whiteway

Cynthia Whitney

Todd Widhelm

Giovanni Widmer

Sivaramesh Wigneshweraraj

Julia Willett

Duncan Wilson

Emma Wilson

Iain Wilson

Michael Wilson

Helen Wing

Wade Winkler

Maureen Wirschell

Christiane Wobus

Katerina Wolf

Benjamin Wolfe

Matthew Wolfgang

Robert Woods

Michael Woodworth

John Woolford

Gerard Wright

Shuqi Xiao

Jianping Xie

Jin-Rong Xu

Ping Xu

Wei Xu

Xin Xu

Chaoyang Xue

Katherine Xue

Kyosuke Yamamoto

Bing Yang

Chunlin Yang

Julianne Yang

Kunlong Yang

Rintaro Yano

Suleyman Yildirim

Peiqi Yin

Jason Young

Mikaeel Young

Jae-Hyuk Yu

Yao Yu

Yunsong Yu

Ze-Chun Yuan

Mark Zabel

Joseph Zackular

Carolyn Zeiner

Lin Zeng

Dan-Dan Zhang

Dongmei Zhang

Hao Zhang

Huajun Zhang

Rong Zhang

Rui Zhang

Wei Zhang

Xiaoli Zhang

Xing Zhang

Yong-Guo Zhang

Yuanwei Zhang

Zhengyi Zhang

Jinping Zhang

Jie Zhao

Kelei Zhao

Kuan Zhao

Weishu Zhao

Zhe Zhao

Aihua Zheng

Chengkun Zheng

Chunfu Zheng

Qi Zheng

Qingfei Zheng

Zhi-Ping Zhong

Hongbo Zhou

Jingwen Zhou

Bofeng Zhu

Honghui Zhu

Ryan Ziels

Sara Zimmer

Franz Georg Zingl

Annelies Zinkernagel

Nikola Zlatkov

Sadri Znaidi

Reza Zolfaghari Emameh

Qi Zou

Jonathan Zuckerman

